# The Venular Side of Cerebral Amyloid Angiopathy: Proof of Concept of a Neglected Issue

**DOI:** 10.3390/biomedicines11102663

**Published:** 2023-09-28

**Authors:** Marialuisa Zedde, Ilaria Grisendi, Federica Assenza, Gabriele Vandelli, Manuela Napoli, Claudio Moratti, Piergiorgio Lochner, David J. Seiffge, Fabrizio Piazza, Franco Valzania, Rosario Pascarella

**Affiliations:** 1 Neurology Unit, Stroke Unit, AUSL-IRCCS di Reggio Emilia, Via Amendola 2, 42122 Reggio Emilia, Italy; 2 Neuroradiology Unit, AUSL-IRCCS di Reggio Emilia, Via Amendola 2, 42122 Reggio Emilia, Italy; 3 Department of Neurology, Saarland University Medical Center, 66421 Homburg, Germany; piergiorgio.lochner@gmail.com; 4 Department of Neurology, Inselspital, Bern University Hospital, University of Bern, 3010 Bern, Switzerland; 5 CAA and AD Translational Research and Biomarkers Laboratory, School of Medicine and Surgery, University of Milano-Bicocca, Via Cadore 48, 20900 Monza, Italy; fabrizio.piazza@unimib.it

**Keywords:** cerebral amyloid angiopathy, CAA, Alzheimer’s disease, AD, cortical angioarchitecture, venule, ascending venule, descending artery, pial network, glymphatic system, perivascular spaces, microbleeds, microinfarction

## Abstract

Small vessel diseases (SVD) is an umbrella term including several entities affecting small arteries, arterioles, capillaries, and venules in the brain. One of the most relevant and prevalent SVDs is cerebral amyloid angiopathy (CAA), whose pathological hallmark is the deposition of amyloid fragments in the walls of small cortical and leptomeningeal vessels. CAA frequently coexists with Alzheimer’s Disease (AD), and both are associated with cerebrovascular events, cognitive impairment, and dementia. CAA and AD share pathophysiological, histopathological and neuroimaging issues. The venular involvement in both diseases has been neglected, although both animal models and human histopathological studies found a deposition of amyloid beta in cortical venules. This review aimed to summarize the available information about venular involvement in CAA, starting from the biological level with the putative pathomechanisms of cerebral damage, passing through the definition of the peculiar angioarchitecture of the human cortex with the functional organization and consequences of cortical arteriolar and venular occlusion, and ending to the hypothesized links between cortical venular involvement and the main neuroimaging markers of the disease.

## 1. Introduction

Cerebral small vessel diseases (SVD) are a relevant cause of ischemic and hemorrhagic stroke and dementia. This category includes different diseases with a common shared point, i.e., the involvement of small vessels in the brain and leptomeninges. The definition of small vessels [1] includes small arterioles, capillaries and venules with a caliber commonly considered under a threshold of 400 µm. Cerebral amyloid angiopathy (CAA) is among the most prevalent SVD, particularly in the elderly. Its hallmark is vessel wall disruption because of the accumulation of amyloid fragments starting from the adventitia of the leptomeningeal and cortical arterioles, capillaries, and venules [2]. CAA is often coexistent with Alzheimer’s Disease (AD), and their pathophysiology shows some common points [3]. One of the key pathophysiological mechanisms shared by neurodegenerative and cerebrovascular diseases is the change in blood-brain barrier (BBB) permeability due to the dysfunction of the neurovascular unit [4,5,6]. This hypothesis has been explored in amyloid-related brain diseases, such as AD and CAA. Indeed, the proposal of the glymphatic system dysfunction leading to protein accumulation because of the impairment of removal has a similar meaning in a more articulated construct [7,8,9,10]. The glymphatic system theory involves a perivascular efflux pathway of interstitial fluid and waste from an arteriole to a venule. The venular side of this dysfunction has been only occasionally investigated.

Nevertheless, a venous vascular disease was described, mainly in histopathological reports, e.g., venous collagenosis in the deep medullary veins [11], as a cause or contributor to leukoaraiosis and white matter hyperintensities (WMHs). Moreover, veins, particularly venules, are included within the small vessels, but the role of amyloid deposits in cortical and pial venules has been poorly investigated, although documented in both animal models [12] and humans [13]. This narrative review aims to describe the anatomical and pathophysiological hypotheses supporting a role for amyloid deposition in cortical and leptomeningeal venules in CAA and AD, highlight neuroimaging hallmarks of the disease, and identify the main open question for future studies. 

## 2. Overview of Vascular Amyloid Deposition in CAA and AD

The main pathological hallmark of CAA is the progressive deposition of Aβ40 and Aβ42 peptides within the wall of the cortical and leptomeningeal small vessels (arterioles, capillaries, and venules) [2]. Aβ40 fragment is the main isoform in vascular amyloid, and Aβ42 fragment is more frequently found in parenchymal plaques; however, Aβ42 is needed to initiate deposition in vessels both in animal models and in humans with CAA [14,15]. The Aβ40:42 ratio of cortical capillaries in tissue specimens is similar to that of parenchymal plaques and different from the one measured in arterial or venular CAA [14,16]. The Aβ42 fragment may then be the initial seed of vascular Aβ, and it deposits later into parenchymal plaques when the disease progresses. Aβ40 fragments are smaller and soluble, and the impairment of the perivascular clearance facilitates their deposition along arteries and veins. This different ratio of amyloid fragments refers to the histopathological definition of type I and type II CAA, respectively, with and without capillary involvement [14]. Vascular Aβ deposition affects the integrity and function of small vessels and, in particular, increases resistance and decreases blood flow, facilitating both microinfarctions and microhemorrhages [17]. This has been demonstrated in the arteriolar and capillary side of CAA and AD but is still neglected on the venular side. Moreover, a dysfunction in the neurovascular unit (NVU) and perivascular transport route has been proposed in CAA and AD, but the venular side of this dysfunction has rarely been addressed. 

## 3. The Venular Side of the NVU and the Glymphatic System

The recently revised concept of neurovasculome [18] represents an evolution of the NVU theory, including the entire extracranial (aortic arch to base of the skull) and intracranial vasculature and associated cells in the skull, brain, and meninges. Limiting the analysis to the venous side, the extracranial veins have a well-defined role in regulating cerebrospinal fluid (CSF) homeostasis and clearance [19]. Within this larger frame, the NVU is the specific neurovascular association occurring at each segment of the neurovasculome [18]. Therefore, the neurovasculome is constituted by multiple NVUs, and they may differ in cell composition depending on the specific vascular segment [20]. These differences were explored in murine models ranging from the pial arterioles to the pial venules through the penetrating arterial branches, capillaries, and ascending venules. The architecture and function of the NVU were investigated mainly in the neocortex, distinguishing the following sequential zones: pial artery/arteriole, penetrating arteriole, arteriole-capillary transition, capillary bed, post-capillary venules, ascending venule and pial vein/venule [18]. Capillaries form a dense tridimensional network throughout the parenchyma and account for >90% of the total length of the vessels in mice [21]. They join into a larger vessel, transition between capillaries and veins and drain into ascending venules, followed by pial venules. Cell types differ in different zones: the arteriolar side contains arterial endothelial cells and arteriolar smooth muscle cells (aSΜC); the venular side contains venous endothelial cells and venous smooth muscle cells (vSΜC). The same cells have different features in arteries and veins. The basement membrane on the vessel and glial sides limits the perivascular space, containing several other cell types, such as fibroblasts and macrophages. Larger arterioles have a more evident perivascular space than smaller arterioles due to the fusion of the glial and pial basement membrane layers as the caliber of the vessel decreases. Pericytes are represented in capillaries and post-capillary veins. Iuxtavascular microglia and oligodendrocyte precursor cells are present from the arteriolar-capillary transition zone to the capillary-venular transition zone. 

NVU regulates cerebral blood flow (CBF) and waste clearance. Cerebrovascular autoregulation is an adaptive process that stabilizes CBF with changing arterial pressure, the main determinant of cerebral perfusion pressure [22]. The arteriolar side of the NVU works as the main site of autoregulation due to the myogenic response of the aSΜCs for guaranteeing the neurovascular coupling. The functions of the NVU are based on the BBB integrity [23], avoiding the paracellular passage of water-soluble molecules through specialized tight junctions among the endothelial cells and allowing molecule-specific exchange through specific influx and efflux transporters. In this way, the BBB assures a restricted permeability [24,25,26,27,28], leaving transcytosis to the post-capillary venules [29], which might represent a point-of-least resistance. Brain Aβ homeostasis is maintained through the endothelial regulation of influx and efflux transporters [5,19]. The waste clearance from the brain parenchyma follows several routes among the BBB, dural lymphatic vessels and along the perivascular spaces [19]. Perivascular clearance received increasing attention because of its proposed role in the pathophysiology of SVD and dementia, particularly amyloid-related brain diseases, such as CAA and AD. According to the glymphatic model, soluble molecules enter from the periarteriolar spaces, where CSF mixes with interstitial fluid (ISF), and drain along perivenular spaces in the same direction as blood flow [30,31].

On the contrary, the intramural periarterial drainage model suggests that solutes within the interstitial fluid drain along the basement membranes of the same arterioles in the opposite direction of blood flow [32,33]. An alternative model proposes that CSF and interstitial fluid mix at the pial surface and soluble waste products are cleared without unidirectional flow [34]. The driving forces moving solutes along the different routes are a crucial issue, and arterial pulsations [35,36,37] and vasomotion [38,39,40,41] were proposed for this role. The translation from animal models to humans is not immediate, and preclinical studies investigating perivascular fluid flow often rely on invasive techniques (including cranial window surgeries and injection of tracers) and the use of anesthesia. Both of them may affect cerebral homeostasis and hemodynamics [41]. 

Among the mechanisms involved in the clearance of small solutes, such as Aβ, the perivascular drainage pathway is the main route in CAA and AD. The perivascular drainage pathway is the movement of small solutes along the ISF and into the perivascular spaces or Virchow-Robin spaces [42]. The two proposed perivascular drainage pathways for Aβ are the intramural periarterial drainage and the glymphatic clearance [43,44]. The intramural periarterial drainage pathway involves the bulk flow of ISF into the basement membranes of capillaries and arteries [45]. In CAA, amyloid fragments usually deposit along SΜs of the tunica media and basement membranes of the tunica adventitia of artery walls, which is the site of the periarterial drainage. This suggests that poor clearance of this drainage pathway contributes to vascular Aβ deposition and CAA. The intramural periarterial drainage pathway does not involve venular function. In addition, ApoE ε4 has also been associated with reduced perivascular drainage of Aβ along the periarterial pathway [19]. The glymphatic clearance pathway involves the periarterial influx of CSF entering the brain, which combines with ISF and moves along the venous perivascular spaces of large cerebral veins [33,44]. Soluble Aβ is one of the solutes that drain along the glymphatic clearance pathway, and recent reports demonstrate this perivenous drainage to be impaired in AD [43,46]. 

Although both arteries and veins are involved in Aβ drainage, there has been controversy regarding the distribution of Aβ within different vessel walls. Pathological studies demonstrated that CAA leads to amyloid deposition along arteries and capillaries, with minimal involvement of veins in AD [47,48]. The main limitation of the human pathological data is the lack of consistent methods used to distinguish between arterioles and venules [49], partially due to brain tissue processing. Therefore, the venous-related pathophysiology should not be neglected in AD. A detailed knowledge of the angioarchitecture of the cerebral cortex may help to understand the role of arteries and veins in these diseases.

## 4. Functional Cortical Angioarchitecture in Animal Models and Humans

The main information about the angioarchitecture of the cerebral cortex comes from animal studies, but old anatomical studies in humans are available, too [50]. Classical studies of the microvascular network [51] are based on Indian ink fillings and scanning electron microscopy of vascular corrosion casts [52,53,54]. However, these studies are far from the actual possibility of imaging the tridimensional vascular network of the neocortex in animal models because of the relatively low resolution [55,56,57]. The most useful anatomical and functional information on cortical angioarchitecture in humans is still provided by studies with Indian ink fillings and scanning electron microscopy of vascular corrosion casts [52]. 

### 4.1. General Organization of the Cortical Angioarchitecture in Humans: The Arterial Side

Arterioles reaching the cortical surface arise from arteries in two ways: directly from the principal trunk at the sulcus surface or with a hidden origin in the depth of the sulcus. The pial arteries have characteristic branching patterns, summarized in Table 1. 

Moreover, at the cortical surface, there are several pial arterial anastomoses with multiple inter-anastomosing arterial sources for each gyrus. Two types of anastomoses are found:

(I). numerous large diameter anastomoses joining arterial branches end to end;
(II). extremely small diameter anastomoses joining two adjacent arteries by a straight course.

Type II anastomoses often join superficial arteries where they penetrate the cortex and form a mesh-like structure known as a pial network, which ensures a robust delivery of blood to the cerebral cortex. The arterioles of the network are attached to the trabeculae connecting the dura and the pia mater and are surrounded by a pial cell lining in the subarachnoid space. On the contrary, the venules underlying the arteries are generally free of arachnoid trabeculae. Once an artery leaves the pial network, it penetrates perpendicularly into the cortex within a perivascular channel [58], limited by a pial cell sheath. The perivascular channel is perforated at the arteriolar level and completely absent in capillaries and veins. Astrocytes define the perivascular or Virchow-Robin space, a clearing route for interstitial solutes, as previously detailed [59]. The cortical arteries sendoff collaterals at different cortical depths, according to the classification scheme by Duvernoy et al. [52], in which a vessel of type 1 would feed/drain superficial cortical layers.

In contrast, higher-order vessel types would feed/drain deeper layers. Some cortical arteries penetrate the entire cortex without collateral until they reach white matter. These classes are not completely separable but appear instead as a continuum. Cortical arteries branch into arterioles and end in the capillary network, finally converging into the venous system. The basic building principle of the cortical vasculature is delivery of blood from the cortical surface, perpendicular penetration into the cortex and, after the capillary passage, drainage back to the surface, where the blood is transported away via the venous system that converges into large sinuses.

### 4.2. General Organization of the Cortical Angioarchitecture in Humans: The Venous Side

The gross description of the venous network [52] is usually done against the flow direction. Then, it starts from the large cortical venous trunks and ends at the emergence at the cortical surface. Collecting venous trunks remain at the surface and are visible for their entire course, while large arteries are usually hidden within the sulcus. Therefore, superficial cortical veins directly join principal collecting trunks without entering the sulcus. Generally, the pial venous network is highly dense and composed of veins larger than corresponding arteries with a slightly angular pathway. It receives many highly ramified branches along its entire course. Superficial veins have two main features:

(I). the cortical venous network may merge into a single trunk at the gyrus center with a star-like appearance (central vein of the gyrus);
(II). the peripheral veins are parallel with respect to each other and flow into a marginal collecting vein along the gyrus edge.

In most cases, peripheral and central dispositions are present on the same gyrus. According to their emergence at the cortex surface, intracortical veins can be divided into large and small-diameter veins. Small-diameter intracortical veins are numerous, have a short superficial course and drain into a large vein at its point of emergence. In most cases, the superficial venous network is anastomotic, and by their anastomoses, the veins form large meshes at the cortical surface. Nevertheless, some venous branching may remain independent of neighboring systems; peripheral or parallel veins, often visible on elongated gyri, may be separated from veins on the opposite side by an avascular zone.

Moreover, some lobules are devoid of notable venous anastomoses. Finally, superficial venous anastomoses are fewer than arterial anastomoses. Generally, the average diameter of pial veins is larger than that of arteries, but it is within the threshold of small vessels caliper [60]. Central veins have an average diameter of 280 to 380 μm, and peripheral veins have an average diameter of 130 μm; even though few, venous anastomoses often have a large diameter, which may reach 180 μm. There are two types of relationships between superficial cortical veins and the arachnoid:

(I). branches at the cortical surface are found under the arterial network and in close contact with the cortex;
(I). there is little contact with the arachnoid layer.

At a pial level, the arterial network covers the venous network with few exceptions. Pial cortical veins are characterized by their large diameters and numerous branches; pial cortical arteries are generally small in diameter and form a network characterized by right-angled branches and large meshes. The arterial and venous networks often condense in a shallow depression that may be found at the gyrus center. These vessels at the lobule center are smaller than the large-diameter arteries and veins within the sulci. In elongated gyri, there is no higher vessel density in the center, but arteries and especially veins on both sides are separated by a poorly vascularized region. It has been reported [52] that the density of superficial vessels varies among different cortical regions: the occipital lobe surface is highly vascularized, whereas a less dense vascular network is found at the top of the hemispheres near the interhemispheric fissure. The measurements of superficial vessel diameters show that veins and their anastomoses are generally larger than arteries.

A narrowing of arteriole diameters at their point of origin on the principal trunk was noted. In contrast to arteries, veins show no change in diameter, but arterioles crossing over veins compress and form a groove in the venous walls, which may slow the blood flow. Annular thickenings of the arachnoid, forming ring-like structures at the junction of cortical veins and the collecting trunk, may also decrease the blood flow. Generally, arteries penetrate, and veins emerge from the cortex at right angles. Penetrating (descending) arteries (DAs) are more numerous than ascending veins (AVs). Large-diameter intracortical veins often emerge from the nervous tissue in a regular pattern. Arteries often penetrate in a ring around each principal vein. 

Several arterial and venous anastomoses at the cortex surface have been described; arterial anastomoses are always present and numerous, and, on the contrary, venous anastomoses vary in number according to the gyri. The question of arterio-venous anastomoses has been debated, and they had not been a convincing demonstration until now. Due to the absence of arterio-venous anastomoses and a capillary network at the cortical surface, blood must flow through the intracortical vascular network before being drained by superficial veins.

There are distinct differences in the ultrastructure between the various types of cerebral blood vessels. Common to arteries, capillaries and veins are the endothelial cell layer and the thin basal membrane. Endothelial cells are coupled by tight junctions and form the BBB that restricts the passage of molecules from the blood to the brain. Whereas capillaries only consist of these two elements: arteries and, to a much lesser extent, veins are covered with a smooth muscle sheath. These smooth muscles regulate vascular resistance by changing the vessel diameter. Pericytes are a very heterogeneous cell type that has a claw-like appearance and are located on the abluminal side of endothelial cells [61]. They also have a contractile capacity, but their role as active regulators of vascular resistance is debated. Furthermore, all small vessels are almost completely covered by astrocytic endfeet [62]. This perivascular astrocytic sheath is thought to play a pivotal role in the transcellular trafficking of metabolites and water and ion exchange at the blood-brain interface.

### 4.3. General Organization of the Cortical Angioarchitecture in Humans: Capillary Network

The diameters of cortical vessels range from around 4 μm (capillaries) to a few tens of microns (arteries/veins) [52]. When looking at the distribution of the calibers, it becomes obvious that the most frequent vessel type is capillary, irrespective of the species [56]. The capillary network can be considered redundant, with a mesh width of approximately 50 μm. This mesh width is probably adjusted to the diffusion constant of oxygen in brain tissue. Although similar vascular network characteristics can be found throughout the cortex, there are variations across the cortical layers and between different cortical areas. In the primary sensory areas, the highest vascular density can be found in layer IV. This is most obvious in the primate primary visual cortex, where layer IVcβ displays an increase in vascular density that is easily detectable [54,63].

When focusing on the pure length as in length density, the largest contribution is provided by the capillaries. However, for other quantities, the contribution of the non-capillaries may be higher, according to their respective dependence on the vessel radius. Due to the volume fraction’s quadratic dependence on the diameter, the contribution of large vessels surpasses that of the capillaries despite their much lower frequency. The vascular density differences between the primary and non-primary areas were also found in the somatosensory and auditory cortex, and the vascular densities of the secondary auditory and somatosensory areas were comparable to those of the extrastriate cortex [51]. Taken together, it seems that animals’ non-primary cortical areas share a similar microvascular architecture. 

### 4.4. Cortical Descending Arteries and Ascending Veins

In humans, Duvernoy et al. [52] state that DAs and AVs of group 4 are the most frequent group of penetrating trees (Table 2).

Typical intracortical arteries can be found in group 4 [52], and their branching from the surface inward has been described as follows in Table 3.

This is the typical appearance of the branching pattern of intracortical arteries, but there are some variations in the different layers of the cortex and unusual aspects. Both issues are out of topic in this review. 

Intracortical veins are included in the same classification by Duvernoy [52], illustrated in Table 2. The venous trunk has a rectilinear orientation in the superficial layers and is slightly angular in deeper layers. Branching is less characteristic than for arteries and, therefore, difficult to describe. Generally, the deeper the venous branching, the longer it becomes (Table 4).

Intracortical veins are characterized by a large number of branches which condense and give the vascular cortical network a bush-like appearance. Triangular enlargements are often present at the junction of small secondary branches and provide a general distinction between venous and arterial branching. As for arteries, there is a huge variation according to group and layer. The most remarkable characteristics of intracortical veins can be seen in group 5 (Table 2), and they should be called the principal veins of the cortex since their size and territory far surpass that of group 5 arteries. The principal vein issues from the white matter and bends in the subcortical zone before passing through the cortex. Along its course through the cortex, the principal vein is similar in appearance to the typical vein of group 4. Only its extremely large diameter differs from other cortical veins. The same type of branching was found in group 4. However, the increase in diameter and length of the branches from superficial towards deep layers of the cortex is even more pronounced; thus, the vascular territory of the principal veins has a conical appearance with its broad base near the white matter. The most superficial branches are found in layer I or at the cortex surface, where they converge towards the point of emergence of the principal vein, and these pial branches drain layers I and II. The deepest branches have an extremely long course, often found in the subcortical zone, and their branching penetrates layers VI and V. The fundamental role of principal veins in the drainage of the cortex. Due to their irregular form, veins in groups 1, 2 and 3 differ from the large veins in groups 4 and 5: in the middle layers of the cortex, veins in group 3 have several branches of origin; their extensive arborization has a bush-like appearance. The venous trunk is often irregular; it is important to note large dilatations at the junction of these branches of origin. The small veins in group 2 are numerous and drain layers III, II and I; their morphology is similar to veins in group 3. The veins in group 1 reach the cortex surface by a short pathway from their origin in layers I and II. For the most part, veins in groups 1 and 2 are independent of each other until reaching the cortex surface; they may, at times, flow into the trunk of veins in groups 4 and 5 and, in this case, become superficial branches. As for arteries, the veins of intermediate length (groups 2 and 3) are the most numerous.

Unfortunately, the technique used by Duvernoy [52] did not allow for reliably measuring the diameters of intracortical veins because fixation and embedding often greatly deform veins due to the thinness of their walls. Principal veins have an average diameter of 80 μm with India ink injections and 120 to 125 μm with methyl methacrylate injections. By this injection method, group V4 veins have an average diameter of 65 μm, 45 μm for group V3, 30 μm for group V2 and 20 μm for group 1. 

### 4.5. Organization of Cortical Arterial and Venous Circulation

The knowledge of intracortical vessel anatomy may help to evaluate cortical circulation [52]. The orientation of arterial and venous branches indicates the existence of 3 cortical blood flows, summarized in Table 5. 

In general, in the superficial zone, the arterial supply is largely of deep origin, whereas venous drainage is superficial. On the contrary, in the deep zone of the cortex, the disposition of long, deep branches of principal veins draining a large territory appears to be common in the central nervous system (perforating cortico-medullary veins).

A regular organization of vessels was found in the human cortex in circular blocks with venous units centered by a principal vein (V5) or smaller veins (V4 or V3) surrounded by an arterial ring. The largest venous units centered by a principal vein (V5) cover a large territory and may be surrounded by several concentric arterial rings. A large number of arteries compared to veins results from this special arrangement: an average of four times the number of arteries as veins reach the middle and deep layers of the cortex. There may sometimes be adjacent principal veins within the same venous unit. It is important to note the uniform disposition of these principal veins. The volume of venous units is quite variable: units centered by intermediate-size veins generally range from 0.75 to 1 mm in diameter, and those centered by principal veins range from I to 4 mm. 

In addition to the overall vascular density, the relative volume fraction occupied by blood vessels of different categories, i.e., arteries, capillaries, and veins, is relevant for many applications. For example, the fraction of blood found in the different vessel types is an essential parameter in compartmental biophysical models. The measurements from the macaque visual cortex estimated that capillaries (defined as vessels with diameter < 8 μm) made up approximately 41% of the total vascular volume fraction [54]. This is similar to an estimate of ~48% obtained from Indian ink-injected sections of the human cortex, which were taken from the fusiform and parahippocampal gyri [64]. While there is a range of microvascular data comparing the relative number of DAs and AVs, no data exist describing blood volume distribution between arterial and venous compartments. Using MRI and PET, Barrett et al. [65] estimated arteries make up ~29% of total blood volume, and veins contribute ~27%, mainly using human and primate data.

Finally, the cortical drainage has a surprisingly regular organization [66,67]. Within grey matter, capillaries of 5 μm diameter and mean length 200 μm drain into venules of 10 to 20 μm diameter. These combine at angles close to 90°, to form larger venules. When a diameter of about 50–100 μm is reached, the venule is termed a principal intracortical vein (PIV) and typically leads directly to the cortical surface at right angles. These veins often extend the cortex’s full depth (about 3 mm), increasing in diameter as they approach the surface, swelled by incoming venules. Once the PIVs, spaced about 300–500 μm apart, reach the surface, they combine to form pial veins in a two-dimensional drainage network on the cortical surface. Like intracortical venules, the pial veins join nearly at right angles. Throughout the arterial circulation, Murray’s Law applies vessels join so that the cube of the diameter of the combined vessel is equal to the sum of the cubes of the diameters of the two branches [68,69]. Assuming that this relationship is maintained in the venous drainage, as confirmed for the retinal circulation [70], at many junctions, the diameter of one tributary is less than 25% of that of the other. Duvernoy’s venograms [71] show that the fine structure of the venous branching is very similar to the larger-scale structure. 

Summarizing the functional organization of the cortical angioarchitecture, Duvernoy [52] described how penetrating venules formed “units” that were surrounded by rings of penetrating arterioles [50]. Although the exact ratio was not specified in their work, a typical penetrating venule appeared to drain blood supplied by ~4–5 penetrating arterioles. This organization of human cortical vasculature implies that occlusion of one penetrating venule would greatly increase the resistance in multiple upstream arterioles. Thus, the human cortical angioarchitecture places penetrating venules at the center of a large perfusion domain, making them a point of vulnerability during cerebrovascular disease [48]. Of further interest is the distribution of DAs concerning each other and to the AVs. The ratio of DAs to AVs is highly species-dependent, and while for primates, there are more DAs than AVs, the opposite trend is observed for rodents (Table 6). The evolutionary basis for these differences remains unclear.

### 4.6. Functional Perspectives on the Consequences of Single Descending Artery or Ascending Vein Occlusion

Some anatomical and functional considerations are useful to explain the differences in the consequences of the occlusion of a single DA vs. AV. Two different issues have relevance for this review in describing the angioarchitecture of the cortex from the venous point of view, i.e., the pial (venous) network and the cortical ascending venules. 

The pial network is located at the cortical surface. The pial arteries distribute blood from the large cerebral arteries to the intracortical vessels, and the pial veins collect it. A robust network topology is of paramount importance for assuring a constant blood supply, and this is achieved by a large number of arterial anastomoses, which, on average, contain four edges [52,75,77]. Overall, the structure of the pial arterial network is comparable to that of a honeycomb with a backbone helping to analyze redundancies [75]. The backbone spans the whole territory of the pial network, even though it comprises only ~11% of the pial arteries. In addition, nearly 75% of all DAs start at a backbone edge. This property further increases the robustness because two different pial vessels can feed those arteries. Occlusion experiments have demonstrated the robustness of the pial network [77], showing that single-vessel occlusion induces a redistribution of flow with the positive effect that all vessels stay perfused.

Nonetheless, the flow rates in individual vessels are significantly affected, and flow reversals, reductions and even increases are observed [77]. The flow is preserved in response to pial artery occlusion in low flux DAs, while it decreases in high flux DAs [75]. This suggests that high flux DAs contain blood reserves that can be redistributed in case of occlusion. The network topology of the pial veins is significantly less studied. Generally, pial veins are larger in diameter than pial arterioles. In the human brain, large pial veins tend to surpass the sulci and remain at the cortical surface. This contrasts with pial arteries, where the main trunk is often within the sulci. Furthermore, pial arteries normally run above pial veins [52].

The DAs deliver blood to the capillary bed over the entire depth of the cortex. Finally, it is collected in the ascending veins (AVs) and returns towards the cortical surface. It is well established that DAs and AVs have a tree-like structure and differ significantly in their penetration depth [52,53,57,64,78,79]. The most widespread classification for penetrating vessels is by Duvernoy [52]. The authors proposed that different groups of DAs are responsible for feeding different cortical layers, as confirmed more than 30 years later by Guibert et al. [80], showing that the depth of the feeding region strongly correlates with the penetration depth of the DA. The DAs belonging to group 4 are the most numerous [52], and the number of branches feeding the capillary bed peaks at cortical layer IV [81]. While this evidence suggests that the DA topology is designed with the major purpose of supplying blood to layer IV, topological characteristics alone do not predict flow. Another issue is the distribution of DAs with respect to each other and the Avs (Table 6). It has been hypothesized that the distribution of DAs correlates with the location of functional neuronal units, but there is conflicting evidence about it. However, it seems likely that each DA is responsible for feeding a specific tissue volume. The occlusion of a single DA in the rat cortex [82] led to an estimate of the feeding region of individual DAs, showing that the infarct volume is proportional to the baseline flux in the occluded DA and, on average, affects a cylindrical volume with a radius of 460 μm and a depth of 1.17 mm. Accurate comparisons are also complex because the DA density increases with the distance to the origin of the middle cerebral artery (ΜCA).

Consequently, the feeding volume is likely to decrease. Additionally, the density of penetrating vessels differs depending on cortical area [74]. The effect of a DA occlusion is apparent up to ten segments from the site of occlusion. 

Even though evidence suggests that an AV occlusion is as critical as a DA occlusion [82], the AVs have received significantly less attention than the DAs. Nguyen et al. [76] hypothesized that if the ratio of DAs to AVs is larger than 1, an AV occlusion is more severe and vice versa. For the marmoset, macaque, and human, where the ratio of DA: AV is greater than 1, the draining region of one AV is significantly larger than the feeding region of one DA [54,78,83]. Furthermore, the drainage volume increases with the diameter of the AV [83], and the diameter of the penetrating vessels is strongly species-dependent. In the mouse, the mean diameter is 11 μm and 9 μm for DAs and AVs, respectively. Opposing trends have been recorded in the human brain; here, the vessel diameter of AVs is larger than that of DAs (65 μm vs. 35 μm for penetrating vessels of group 4) [52].

Moreover, the mean vessel diameter increases with the penetration depth of the DA/AV. Interestingly, in contrast to the DAs, the number of AV offshoots decreases with cortical depth. Relatively little is known about the relative placement of DAs and AVs. Based on the analysis of the microvascular network from the mouse cortex, a rhombic lattice where one DA is encircled by six AVs was suggested [61]. A comparable concept was proposed in humans [52], stating that a “vascular unit” in the human brain consists of an AV surrounded by several DAs (in the human brain, the DA: AV ratio is approximately 2). From a sole fluid dynamical point of view, it is plausible that the AVs/DAs are fed equally by the central DAs/AVs, but Guibert et al. [80] state that 61.5% of the flow of one DA is drained into one AV and Schmid et al. [81] observe that 72% of blood drained by an AV originate from one DA. Nonetheless, although there seems to be a preferential AV for every DA, the DAs and AVs are highly interconnected: in the marmoset, one DA is connected to 22 AVs [80]. 

The capillary bed has a mesh-like structure, generally homogeneous and highly interconnected. Hence, its topology is significantly more difficult to analyze than the pial vessels or the penetrating trees. For each capillary starting point, there are, on average, eight different flow pathways leading from DA to AV. Still, for more than 50% of all capillary starting points, a preferential path is chosen with a frequency >50% [81]. The capillary starting point is strongly correlated to the capillary endpoint. It seems likely that the capillary bed is designed to encourage the in-plane (i.e., parallel to the surface) motion of red blood cells. As for the pial and penetrating vessels, occlusion experiments have been performed to assess the overall robustness of the capillary bed in the distribution of flow [82,84]. Indeed, the impact of a microvessel occlusion is minimal, and the flux recovered to 45% of its baseline value by three branches downstream of the occlusion. The robustness of the capillary bed can be explained by its mesh-like structure, which is beneficial for an efficient redistribution of flow: 63% of all capillaries are fed by more than one DA, and hence, a redundancy towards DA occlusion persists as well [80]. Interestingly, the robustness of the capillary bed towards DA occlusion increases with depth. 

The pial network is responsible for a robust blood supply to different tissue regions. Furthermore, it has been hypothesized that it redistributes blood during neuronal activation [82,85]. Despite differences in methodology and/or species, several studies agree that multiple pial arteries alter their diameter in response to neuronal activation; the largest changes are located close to the center of activation (CoA), and the amplitude of the relative diameter change decreases with the distance from the CoA [51]. Positive diameter changes have been observed up to 3 mm away from the CoA; however, for distances >2 mm, the vascular response is predominantly negative (i.e., more constrictions than dilations [85]. For many pial arteries, there is a period of constriction after stimulus cessation, for reasons not currently understood, and the amplitude of constriction is significantly smaller than that for dilation. The DAs feed the capillary bed with blood from the cortical surface. As they pose the “bottleneck of perfusion” [84], it seems likely that they are also ideally placed for a localized increase in blood flow. Roughly half of the monitored DAs responded to neuronal activation [86], but their distribution with respect to the CoA is unknown. Recent studies reported that the dilation of DAs is initiated deep in the cortex (measurements up to 0.9 mm) and propagates towards the cortical surface, with approximately 50% of all responding DAs experiencing a post-stimulus constriction phase [87]. 

As capillaries are the vessels most proximal to the largest part of the tissue, it seems plausible that they may play a role in the up-regulation of flow, oxygen, and energy substrate delivery. However, there is relatively little data available, and it is challenging to differentiate between effects resulting from arteriole and capillary dilation in vivo. Hall et al. [86] demonstrated that capillaries dilate on average 1.4 s before arterioles, are more likely to respond in the vicinity of pericytes (50% vs. 22% response frequency), and the frequency of responding capillaries decreases with branching order. As DAs, capillaries located deep in the cortex dilate earlier than the ones close to the cortical surface [88]. 

No active dilation of venules and veins has been reported; however, there are conflicting reports about the presence and/or significance of passive dilation of venules and veins, and this issue may be of interest in venular involvement by SVD in general and CAA in particular. For example, direct optical measurements of venous diameter have shown either no increase [89] or very small increases [90]. In contrast, Magnetic Resonance Imaging (MRI)-based approaches have reported considerable increases in venous blood volume [91,92]. These discrepancies could be explained by differences in stimulation length (typically short in optical imaging experiments but long in MRI experiments) and by the fact that even relatively small changes in capillary and/or venous diameters can lead to large changes in venous CBV [65]. This result suggests that, for brief stimulation, dilation of arteries and arterioles contributes to the majority of blood volume increases; however, dilation of post-arteriolar vessels is relevant during longer stimulation (>10 s).

Summarizing the angioarchitecture of cortical veins, typical penetrating venules drain the blood supplied by 4 to 5 penetrating arterioles [51,52]. Due to these anatomical features of the cortical vasculature, stenosis or occlusion of one penetrating venule can increase resistance in multiple upstream arterioles.

## 5. Evidence of Venular Amyloid Deposition

The cerebral venules, particularly in the cortex and pial surface, received scarce or no attention in understanding the mechanisms involved in CAA and, in association or not, AD. Recently, there has been increased importance in studying the pathology of veins and venules, not only in AD or CAA but also in SVD and dementia. Venous pathology has been shown to contribute to vascular dysfunction in AD, resulting in WMHs and microinfarcts and potentially induction of ischemia [51,93,94]. Few reports demonstrated a pathological role for veins in CAA and AD [95,96,97]. One of the most controversial issues is the documentation of venular amyloid, i.e., the deposition of Aβ within cortical and pial veins and venules. 

The strongest evidence about amyloid accumulation in the venular walls comes from in vivo animal models. The APP + PS1 rat model [98] shows severe CAA, Aβ plaque formation and behavioral changes, where Aβ deposits were documented in leptomeningeal and cortical arteries, cortical capillaries, as well as severe deposition in the leptomeningeal and cortical veins using immunohistochemical analyses. Another model is the TgF344-AD rat [99], which overexpressed human Swedish APP and PS1 with exon 9 excised. TgF344-AD rats recapitulate AD progression in humans, including age-dependent cerebral amyloidosis, CAA, tau pathology, neuronal loss, and cognitive dysfunction [99]. In the same rat AD model, Joo and colleagues [100] studied early neurovascular dysfunction, demonstrating that vascular Aβ deposition was not exclusive to arterioles and can be found in venules to a lesser degree at nine months of age. The topography of these amyloid deposits is quite distinct from deposition in arteries: venous Aβ deposits appear more globular and have a patchy distribution, whereas arteriolar Aβ possesses a ‘double-barreling’ morphology, forming striped rings, which is in correspondence with previous literature in humans too [101,102,103]. However, significantly less amyloid deposition in veins and venules relative to arteries and arterioles was documented [100], raising the possibility of an age-dependent association and leaving room to study the contribution of venular deposition to vascular dysfunction in AD, expressed by white matter hyperintensities and microinfarcts [51,93].

Further findings on the same rat model [12] using mass spectrometry analyses demonstrated the presence of Aβ in samples of isolated cortical parenchymal plaques, arteriolar and venular walls, and proteins known to be associated with AD. Histopathology confirmed the hypothesis of a significant age effect for arteriolar and venular amyloid accumulation, adding a proposal of topographical progression. Indeed, the accumulation is initiated in the somatosensory cortex, followed by the motor and cingulate cortex. Moreover, significant arteriolar amyloid accumulates relative to venular amyloid deposition in AD progression. Collagenosis of deep medullary veins has been documented in humans, and it may be a contributor to WMHs, but no amyloid-beta deposition was found at this level, and the aim of this review is the involvement of cortical and leptomeningeal venules and not the collagenosis of the deep medullary veins. In mouse models, the impairment and/or suppression of both the periarterial drainage pathway and the glymphatic clearance pathway in AD [46,104,105].

Interestingly, this impairment is on the arteriolar and capillary side, not the venous side [45,104]. However, in the Tg2576 AD mice, only rats of 22 months exhibit signs of impairment in the perivascular route on the venous side [104], as for increasingly impaired glymphatic drainage with aging [46,105]. Only one model (APP/PS1/Cx3cr1 mice) demonstrated early venular amyloid deposition (at five months and nine months of age) together with arteriolar deposition [102]. The authors proposed that the Aβ deposits in veins precede arterial Aβ deposition, which is contrary to previous findings, and demonstrated preferential adherence of monocytes to surrounding Aβ-laden veins, but not arteries, as removal route through the internalization of venular amyloid by these monocytes [102]. 

The proposed reasons for lesser amyloid deposition in veins than in arteries in animal models of AD are two:

(I). the lack of highly organized SMCs in veins and venules, resulting in less substrates for Aβ deposition and
(II). monocytes that exclusively adhere to veins, clearing Aβ as it deposits along the venular walls [16,42,106].

Unfortunately, there are reports that do not distinguish between arteries and veins [107,108,109,110,111] or exclusively report CAA in arteries [112,113]. 

Besides the animal models, we have small clinical evidence about venular amyloid deposition, reported in individual cases as early as the 1980s [114]. In a case series of 45 patients, the proportion of Aβ deposition in each vessel was recorded according to a severity measurement, 0–4, now termed the Mountjoy scale [95,114], and the presence of Aβ deposits in veins was demonstrated in 21 patients with one case having severe amyloid deposition in veins [114]. Weller and colleagues also demonstrated Aβ deposition in veins, although to a lesser extent than arterial Aβ deposition [115,116]. One case study examined 17 AD brains to determine Aβ deposition in accordance with perivascular drainage. The leptomeningeal vessels were stripped from the surface of the brain and were stained with Thioflavin S to quantify amyloid deposition in 681 arteries and 352 veins. A deposition demonstrated a 5:1 distribution in arteries compared to veins, with approximately 4.5% of veins positive for amyloid [116]. The same research group observed Aβ deposition in the walls of veins in the presence of severe CAA associated with AD [115]. As very few Aβ deposits were detected in these veins, Weller and colleagues considered these deposits rare, attributing the CAA pathology to impaired clearance of the periarterial drainage pathway [116].

Summarizing the hypotheses raised by preclinical and clinical evidence, venular Aβ, even if rarer than the arterial one, can impair glymphatic clearance, contribute to Aβ deposition, and exacerbate CAA and AD progression. On the contrary, a neuropathological study of 69 human autopsy brains assessed the distribution of Aβ deposition across different vessels. It is associated with pathological features such as CAA severity and AD-related Aβ pathology [96]. Two types of CAA were identified, CAA Type 1 and CAA Type 2, which were associated with the presence and absence of capillary Aβ deposition, respectively. An important observation in this study was that both CAA types showed Aβ deposition in leptomeningeal and cortical veins, suggesting that CAA is not exclusive to arteries but also in veins and differentially in capillaries [96]. 

A recent paper examined the potential venular involvement in intracerebral hemorrhages [95] in 41 patients showing the presence of CAA, graded by the Vonsattel scale [117] and the Mountjoy scale [114]. 33 out of 41 patients had Aβ deposits in veins, 15 of which showed severe CAA, 13 showed moderate CAA, and the remaining five showed mild CAA [95]. These data support the hypothesis that dysfunction of either the periarterial or the perivenous pathways would shift clearance to the alternate path and strain existing mechanisms, potentially leading to increased deposition in that blood vessel.

## 6. Potential Contribution of the Venular Involvement to Neuroimaging Markers of CAA

Pre-clinical data have confirmed that venular occlusion causes microinfarcts that are remarkably similar to those found in clinic-pathological human studies [118]. The vascular architecture of the human cortex further suggests that each penetrating venule could be a locus of vulnerability for perfusion since multiple arterioles rely on a single venule for drainage. Thickening of venular walls, leukocyte adhesion, capillary pathology, and hypercoagulability caused by amyloid b may cooperate to increase blood flow resistance and venular thrombus. When considering these factors in the context of SVD and cerebral hypoperfusion, we see a potential for venules to become occluded. There is very limited data on the relationship between venular pathology and microinfarct burden in humans. Novel approaches to image vasculature of post-mortem tissues in 3D and recent advances in ultrahigh-field MRI may aid.

A different approach has been performed using high-field MRI in evaluating deep medullary veins tortuosity as a marker for SVD [119]. This application, as the evaluation of collagenosis of the deep medullary veins [11,94,97] and the measurement of venous oxygen levels [120], is outside the aims of this review, and they will not be specifically addressed. Moreover, superficial cortical veins (SCVs) were evaluated using MRI in a study on 344 healthy volunteers aiming to establish a relation between cognition and the diameter, length, or tortuosity index of the SCVs in the bilateral cerebral hemispheres without success [121].

Among the CAA MRI markers outlined in the STRIVE 2.0 [58], there is little or no evidence of venous connection, and three were selected to address this issue: cortical microinfarcts, microbleeds and enlarged perivascular spaces. There are several other MRI markers of SVD [122], e.g., white matter hyperintensities, lacunar infarcts, DWI positive microinfarctions and cerebral atrophy, but no direct relation with a cortical venular involvement has been addressed in the literature until now.

### 6.1. Cortical Microinfarcts

**Figure 1:**
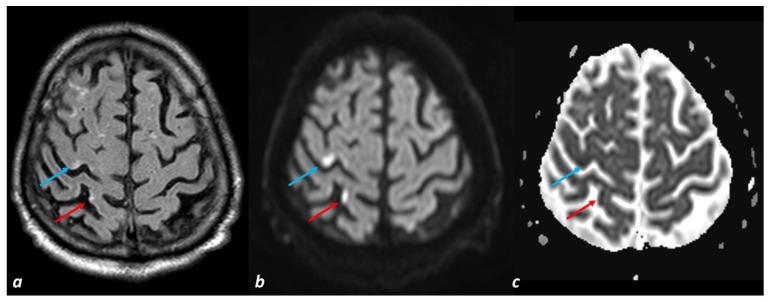
Acute cortical microinfarctions (red and blue arrows) on MRI (axial FLAIR sequence in (**a**), DWI sequence in (**b**) and ADC map in (**c**) in a patient with CAA-related inflammation.

Cerebral microinfarcts are small (0.05–3 mm in diameter) ischemic lesions that can be found nearly everywhere in the human brain [118,123,124]. A meta-analysis of seven large clinic-pathological studies revealed that individuals who died with dementia were nearly twice as likely to have microinfarcts as individuals who died without dementia [119]. Microinfarcts may elicit secondary degeneration of anatomically connected brain regions, as occurs for larger infarcts [125]. Cortical microinfarcts are a marker of SVD and CAA, and they may have different etiological mechanisms (emboli from the heart or large arteries, local thrombus formation in diseased small vessels, and cerebral hypoperfusion) in comparison with subcortical infarcts. An example of cortical microinfarctions is illustrated in Figure 1. 

The potential role of venular disease in CAA and cortical microinfarcts has not been systematically addressed, but it may have some theoretical support. The disease process affecting small venules generally differs from the one affecting arterioles. The animal model allows us to dynamically evaluate how a cortical microinfarct develops with perturbation of cortical microvascular flow [126]. The pial surface vasculature of the cerebral cortex is a highly redundant network, allowing the maintenance of blood flow if one pial arteriole is occluded by rapidly re-routing through anastomotic connections [75,77]. In contrast, there are no anastomoses between penetrating arterioles, which descend from the pial arterioles to perfuse columns of cortical tissue [84]. This lack of collateralization makes penetrating arterioles vulnerable to cortical perfusion. In vivo, photothrombotic occlusion of single penetrating arterioles results in ischemic lesions with remarkable similarity to a subset of human cortical microinfarcts with respect to their location, shape, and absolute volume [82,127]. These similarities exist between rodent and human microinfarcts because the perfusion domains of penetrating arterioles are comparable between species despite an approximately two-fold greater thickness of the human cortex than the rodent cortex. That is, mouse cortical vasculature is closer to a “cropped”, rather than “scaled”, version of human vasculature [64].

The venular network of the cortex mirrors the arteriolar one with a similar structure. As with pial arterioles, pial venules are resilient to localized clots because flow can be efficiently re-directed through anastomotic connections [76]. However, blood emerging through the brain capillaries coalesces into penetrating venules that form a bottleneck in perfusion, as seen with penetrating arterioles. The rodent cortex has ~2–3 times as many penetrating venules as penetrating arterioles [66,82], indicating that each penetrating venule transports only a fraction of the blood carried by a penetrating arteriole. One, therefore, expects that loss of flow through one penetrating venule will produce an infarct smaller than that generated by arteriole occlusion. Surprisingly, occlusion of single penetrating venules generated microinfarcts that were indistinguishable from those caused by penetrating arteriole blockade [82,127,128]. By examining microvascular flow in vivo after venule occlusion, the main finding is that the loss of flow through one penetrating venule led to gradual stagnation and thrombosis of upstream penetrating arterioles and recruitment of the arteriolar perfusion domain into the microinfarct core [129]. Thus, the arterio-venous system acts as a single unit to route blood through the capillary bed, and loss of flow through either penetrating arterioles or penetrating venules produces microinfarcts of comparable size. 

The translation of findings from mouse to human should be done cautiously because the angioarchitecture differs between mouse and human. In the human cortex, there are more penetrating arterioles than penetrating venules in the human cortex, which is the inverse of [76,82,128].

The main focus of our review is the cortical venous involvement, but few information may be driven by subcortical/medullary veins examination in amyloid-related cerebral diseases. A commonly reported venous abnormality is increased tortuosity. Using 7T MRI, investigators observed that patients with mild cognitive impairment and early Alzheimer’s disease had more tortuous deep medullary veins than age-matched controls [129]. Another study showed that healthy middle-aged carriers of the APOE ε4 allele had more tortuous subcortical venules than carriers of other APOE alleles [119]. A higher number of microinfarcts has been reported in deeper nuclei of APOE ε4 carriers (caudate, putamen, globus pallidus, and thalamus), and venous tortuosity may be involved in this pathology [130]. Venule tortuosity was also examined in the TgCRND8 mouse model of Alzheimer’s disease, but no difference was found between transgenic and control mice [93,103]. However, mural cell defects and blunted dilatory responses to hypercapnia were observed with cortical venules in TgCRND8 mice [93] and, more recently, in the TgF344-AD rat model [100]. Whether these rodent models develop spontaneous cortical microinfarcts has not been examined.

Other mechanisms may converge with venule pathology to induce venular obstruction and microinfarcts. One such mechanism is the hypercoagulable state produced by contact between amyloid β and clotting factors, such as factor XII, leading to augmented fibrinogen cleavage in patients and mice with CAA [131,132]. It is conceivable for vascular amyloid and blood-borne clotting factors to interact through an impaired blood-brain barrier, which induces a hypercoagulable state that contributes to microinfarcts. The activation of clotting factors by vascular amyloid may not be potent enough to produce thrombi in fast-flowing arterioles but could promote thrombosis of downstream venules. Another potential factor that can promote venular occlusion is capillary pathology. A role for capillary pathology in the development of dementia has been widely postulated [133,134], given reductions in capillary density in cortex and white matter [135,136]. Direct amyloid β deposition has also been reported near deformed capillaries in patients with AD [137]. Further, as mentioned above, collagenosis occurs in capillary-sized vessels of periventricular tissues during leukoaraiosis [94]. 

Venules are also the primary locus of leukocyte adhesion and entry into the brain under inflammatory conditions [138]. In vivo imaging of animal models of Alzheimer’s disease has shown that pro-inflammatory molecules, such as amyloid β and endothelial dysfunction, promote leukocyte adhesion and entry around venules [102,139]. Furthermore, animal studies have shown that cerebral hypoperfusion resulting from arterial stenosis, a common scenario in vascular cognitive impairment [140], can elicit marked leukocyte adhesion in venules and capillaries [141]. Increased leukocyte adhesion puts venules in a dangerous position, as reactive oxygen species and proteolytic enzymes derived from leukocytes can damage endothelial cells and induce clotting [142]. During hypoperfusion, sluggish flow in venules may arise in ‘watershed’ regions between major cerebral arteries, where perfusion pressure is lowest, and can further reinforce leukocyte adherence and clotting. Indeed, microinfarcts have been reported to be denser in watershed zones of individuals with AD [143].

To date, there have been few studies that have considered venule pathology as a source for microinfarcts. This may, in part, be due to difficulties in differentiating between venules with thickened walls and arteriole hyalinization using routine stains such as hematoxylin and eosin [97,136]. Methods to unambiguously identify venules would be important in future studies on microinfarcts and their spatial overlap with venule pathology. Incorporating additional stains to differentiate arterioles from venules would be key, including alkaline phosphatase [97], a-smooth muscle actin [94], or Alexa 633 hydrazide, which labels elastin in arteries and large arterioles [144]. However, these approaches are not without limitations, as smooth muscle cells and elastin can degenerate during small vessel disease, potentially leaving nothing to stain. Another way to distinguish between small arterioles and venules ex vivo is to follow their connections to larger upstream arterioles or venules where vessel type can be more easily distinguished by morphological and staining features. While this is difficult with thin tissue sections, new tissue clearing and optical imaging protocols [145,146,147,148] allow one to visualize the vascular network in larger volumes [149]. Furthermore, this approach makes it possible to quantify changes in vascular branch patterns and vessel tortuosity.

The structure and function of small venules can also be examined in vivo with ultra-high field MRI. As mentioned above, venule structure in deeper tissues has been examined with susceptibility-weighted imaging [119,129]. Novel methods are also emerging for measuring blood flow velocity and pulsatility in very small cortical and subcortical perforating vessels [150]. Complementing clinical studies are preclinical techniques with impressive spatiotemporal resolution for imaging microvasculature in vivo. Multi-photon microscopy has been used to study cortical venule structure [93] and leukocyte adhesion in models of Alzheimer’s disease [102]. Ultrafast ultrasound imaging allows rapid, non-invasive assessment of arterioles and venules down to <10 µm in diameter in both cortical and deep brain regions [151]. Furthermore, fMRI has achieved resolutions necessary to visualize hemodynamics in individual cortical penetrating arterioles and venules [152]. These techniques can be used to understand the vascular basis of microinfarcts in animal models that develop microinfarcts spontaneously [153,154,155].

Recently, blockage of a single venule in mice vastly impaired cerebrovascular structure and function [50,126]. Acute venular occlusion in the mouse cortex decreases blood flow, increases vessel diameter, impairs local neuronal function and neurovascular coupling, induces severe hypoxia, and can cause somatosensory behavioral deficits [76,82,127]. Interestingly, even though mice have 2–3x more cortical penetrating venules than arterioles, the occlusion of a single venule had a profound impact, indicating the potentially compounding effect of venular amyloid. Conversely, humans have less venules for every arteriole [76], suggesting a greater strain on each venule. Therefore, with the associated collagenosis and vascular impairments in AD, venular amyloid likely accelerates disease progression.

### 6.2. Enlarged Perivascular Spaces

**Figure 2:**
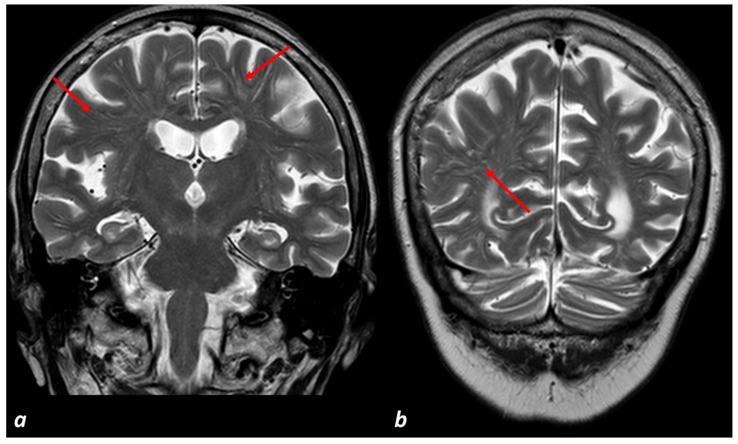
Multiple EPVSs in the CSO and the subcortical WM in T2W-MRI in the coronal plane at the hippocampi (**a**) and occipital poles (**b**) level. The red arrows point to the EPVSs.

Perivascular spaces (PVS) are fluid-filled spaces surrounding small perforating brain blood vessels [58]. They may be part of the glymphatic system [42] and be necessary for brain fluid drainage. Indeed, soluble Aβ in the ISF clears from the brain within arterial and venous perivascular spaces surrounding small cerebral vessels. Flow through these fluid-filled compartments is impeded by perivascular accumulation of cellular debris and amyloid and collagen aggregates on vessel walls. When enlarged, PVS are visible on T2-weighted and T1-weighted MRI as round or linear hyperintensities/hypointensities, respectively, primarily in the basal ganglia and centrum semiovale (CSO). Enlarged perivascular spaces (EPVSs) are a relevant hallmark of SVD in general and CAA [58], being present in AD too. Their relevance in CAA led to the inclusion of the non-hemorrhagic neuroimaging markers incorporated into the new proposed diagnostic criteria Boston 2.0 [156]. An example of EPVSs in a patient followed up for AD is illustrated in Figure 2. 

The drainage routes from the brain involve a para- and perivascular system, where an arterial and venous component have been standardized, mostly in rodents. Theoretically, EPVSs may surround both an artery and a vein, but there are no efficient ways to distinguish these two components, particularly using neuroimaging techniques. Deoxygenated venous blood provides intrinsic contrast on gradient echo and susceptibility-weighted imaging sequences; therefore, vessels visible on these sequences are suspected to be venular. This technique investigated a spatial relationship between suspected venules and PVS to determine associations between venules and patient demographics, risk factors, SVD features, cerebral microvessel dysfunction, and retinal venules in patients with SVD [157]. The main limitation of this study for this review is the recruited population, including patients with lacunar or minor nondisabling ischemic stroke and not specifically patients with CAA. Considering this limitation, only 81 venules had overlapping PVS in all 67 patients (mean percent of total venules that overlapped with PVS, 4.6%; range, 0–18%). Lower venular count was associated with increased pulsatility in the sagittal (β = −0.425 [95% CI, −0.754 to −0.096]) and transverse (β = −0.406 [95% CI, −0.712 to −0.100]) sinuses. The study’s final results are that different locations and infrequent overlap exist between suspected venules and PVS on 3T MRI, suggesting that most venules and MRI-visible PVS are not spatially related. A similar finding in an overlapping population comes from a 7T MRI study [129], and a pathology study [158] also found little venule-PVS overlap, suggesting that MRI-visible PVS in humans might be periarteriolar. 

### 6.3. Microbleeds

**Figure 3:**
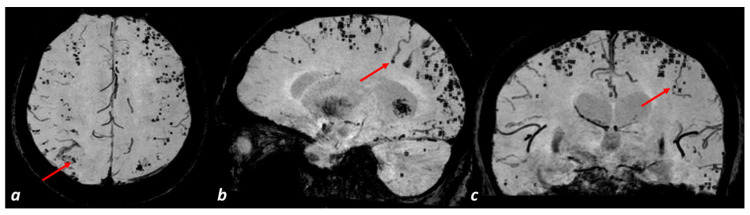
Multiple cortical MBs in a patient with CAA in SWI-MRI reconstructed in axial (**a**) sagittal (**b**) and coronal (**c**) plane. The red arrows point to the potential connection of MBs with cortical venules.

Cerebral microbleeds (MBs) are a typical neuroimaging marker of SVD, defined as small hypointense round lesions visible on T2*-weighted MRI and susceptibility-weighted imaging (SWI) [14,159]. Histopathologic correlation studies claim that the majority of MBs seen on MRI are acute, subacute, or chronic small focal lesions of accumulating intact erythrocytes or hemosiderin [160,161] resulting from leakage or rupture of a small vessel. The neglected involvement of venules in SVD and CAA suggests that MBs mainly derive from the arterial side of the small vessels [162]. However, as suggested in the previous sections, the role of small veins in developing MBs, caused by SVD-related venous pathology is also conceivable. A potential example is shown in Figure 3. 

A single study [163] examined quantitative susceptibility mapping (QSM)—based venograms at submillimeter resolution by using 7T MRI in a cohort of patients with SVD and elderly controls aiming to explore the in vivo relationship between small veins and MBs. This study assessed MBs with a direct link to a vein in the QSM sequence. They were defined as MBs with venous connection (MBven) if the connection was visible at least on one slice. A total of 96 MBven were found in the cohort, representing 14% of all detected MBs on 7T QSM (*n* = 674). Of all participants, 19 (37%) showed MBven, including 7 CAA (88% of all CAA cases), 2 HA (40%), seven mixed MBs (100%), and three controls (10%). The prevalence of MBven in MB presenting controls was consequently 12% (3/25). The vast majority of MBven (87%, *n* = 83) were observed in lobar locations, and all of them were cortical. Patients with CAA showed significantly more lobar MBven than controls (median 3.5 vs. 0; r = 0.57, *p* = 0.002). The distribution of MBs with venous connection in this study was predominantly lobar (87% of all venous MBs), and all of these MBs were located in the cortex. Conversely, only 5% of all venous MBs were found in deep and 8% in infratentorial regions. Furthermore, patients with CAA presented the highest ratio of venous MBs to total MBs (19%). These findings could suggest a relation to CAA pathology in that the association between small veins and MBs could involve vascular Aβ pathology [164] or the related vessel wall remodeling [165]. Pathologic studies that investigated MBs so far have not systematically distinguished whether the ruptured vessel was a vein or an artery [159]. A report of a single patient [166] has been recently published, showing the topographical association between cortical microbleeds and cortical venules, as well as cortical EPVSs, but one of the main limitations is that the patient had CAA-related inflammation, so some of the neuroimaging markers may be different from the expected in CAA.

## 7. Conclusions

Although there is histopathological documentation of venular accumulation of amyloid fragments in both human and animal models, the role of the venous network and venous dysfunction induced by amyloid accumulation in CAA is still under definition. Some information provided by studies on cortical angioarchitecture, even in humans, can provide the basis for formulating hypotheses on the consequences of functional alterations of the cortical venous circulation and even the occlusion of a single AV. How much these pathophysiological hypotheses can be translated into practice is currently difficult to define. Similarly, the role of the alteration of cortical venous hemodynamics in the development and progression of CAA cannot be excluded, as well as in the determinism of some of the neuroimaging markers of the disease, with particular attention to cortical microinfarcts. 

Moreover, no information is available on the differential role of venules in deep and lobar intracerebral hemorrhage (ICH), whose etiology, natural history and prognosis are different [167], being lobar ICH more frequently CAA-related than deep ICH. A further limitation is the pathological documentation that roughly half of spontaneous primary lobar ICH does not have evidence of amyloid deposition in the brain vessels [168]. Arteriovenous malformations, dural arteriovenous fistula and cavernous angioma, and cerebral venous thrombosis are part of the differential diagnosis of macrovascular causes in lobar ICH in routine clinical practice. These limitations represent further research in assessing the venular role in SVD in general and in CAA in particular. In conclusion, more research is needed to refine these hypotheses and acquire strong results. High-field MRI studies are promising for this purpose, but a pathological confirmation of new associations is mandatory.

## Figures and Tables

**Table 1:** Type of branching patterns of pial arteries according to Duvenoys [52].

Branching Type	Description
I	A central artery divides into numerous and highly sinuous branches at the center of the gyrus surface.
II	Peripheral arteries cover the gyrus as a succession of straight segments and angles. The branches entering the cortex and the anastomotic vessels joining the adjacent arteriole arise from each angle.
III	An arteriole arising from the concavity of an arterial trunk divides into several branches, which diverge and vascularize the entire surface of a lobule.
IV	A long-course cortical arteriole with progressively decreasing diameter at the cortex surface ends in the vascular network of the molecular layer of the cortex.

**Table 2:** Classification of penetrating vessels based on their penetration depth by Duvernoy [52]. The classification is valid for DAs and AVs (except for group 6, which only exists for DAs). All penetrating trees, except for group 6, have offshoots along their depth.

Vessel Group	1	2	3	4	5	6
Penetration depth (cortical layer)	I–II	IIIa	IIIc–Va	VI	down to WM	down to WM without branching

WM: white matter.

**Table 3:** Sequential branching of DAs, according to Duvernoy [52].

Branching	Description
(1) Primary or superficial branches	- They originate directly following the trunk’s penetration and spread out parallel to the surface in the superficial layers of the cortex (layers I and II). - Some branches remain within these layers while others penetrate, parallel to each other, into layer III.
(2) Secondary or intermediate branches	- They are characterized by a recurrent course, giving the artery a candelabra-like appearance. - After originating in the deep part of the III or adjacent IV layers, most branches turn towards the surface, end in layers I and II, and divide into several parallel branches in layer III. - Secondary vessels which arise from recurrent arteries may extend in a counterflow direction. - Often, branches arise from the top of the curve of the recurrent branch and penetrate deeper within the cortex, where they contribute to the vascularization of layers IV. - Recurrent branches also arise from the principal trunk at different levels. Most originate in layers III and IV but may sometimes originate in layers V and VI.
(3) Tertiary deep branches	They are frequent and arise at acute angles from the principal trunk and reach the middle (IV) and deep (V and VI) layers of the cortex, where they immediately branch out.
(4) Terminal trunk	It maintains the artery’s general orientation, and it is often very small, ending in layers V and VI of the cortex.

**Table 4:** Sequential branching of AVs, according to Duvernoy [52].

Branching	Description
(1) Primary superficial branches	They are small in size; they flow into the trunk after a short course parallel to the surface in layers I and II or even at the cortex surface.
(2) Secondary or intermediate branches	They are small and few and flow into the trunk, often at an acute angle, in layer III.
(3) Tertiary or deep branches	They flow into the venous trunk beginning in layer IV; these branches are large and numerous and often flow into the main trunk at right angles

**Table 5:** Organization of cortical circulation by zones, according to Duvernoy [52].

Cortical Zone	Description
(1) Deep part of the cortex (layers V–VI)	- Long branches of the main veins located between the cortex and white matter drain the deepest layers of the cortex. - The arteries primarily come from more superficial zones (layers IV and V).
(2) Middle part of the cortex (layer III)	- Arteries and veins quickly divide into numerous branches. - The bush-like venous branches are surrounded by arterial ramifications.
(3) Superficial part of the cortex (layers IIIa-III b-II- I)	- The arterial supply arises from recurrent arteries originating in the middle part of the cortex and curving towards the surface. - The venous drainage is superficial, and it is composed of a large number of type VI and V2 venules, which, following a short course from layers I and II, flow into the pial vessels.

**Table 6:** Average ratios of DAs to AVs, average number of DAs and AVs for different species. References: Human [64,72], Monkey [52,55,73,74], Rat [75,76], Mouse [61,75].

Species	Human	Monkey	Rat	Mouse
Ratio DA:AV	2.2:1	2.1:1	1:1.8	1:3.0
DAs per mm^2^	1.0	7.9	8.3	3.9
AVs per mm^2^	0.5	3.6	10.3	-

## Data Availability

Not applicable.
